# Serial heart rhythm complexity changes in patients with anterior wall ST segment elevation myocardial infarction

**DOI:** 10.1038/srep43507

**Published:** 2017-03-02

**Authors:** Hung-Chih Chiu, Hsi-Pin Ma, Chen Lin, Men-Tzung Lo, Lian-Yu Lin, Cho-Kai Wu, Jiun-Yang Chiang, Jen-Kuang Lee, Chi-Sheng Hung, Tzung-Dau Wang, Li-Yu Daisy Liu, Yi-Lwun Ho, Yen-Hung Lin, Chung-Kang Peng

**Affiliations:** 1 Department of Electrical Engineering, National Tsing Hua University, Hsinchu, Taiwan; 2 Department of Biomedical Sciences and Engineering, National Central University, Taoyuan, Taiwan; 3 Division of Cardiology, Department of Internal Medicine, National Taiwan University Hospital and National Taiwan University College of Medicine, Taipei, Taiwan; 4 Division of Cardiology, Department of Internal Medicine, National Taiwan University Hospital Hsin-Chu Branch, Hsin-Chu, Taiwan; 5 Department of Agronomy, Biometry Division, National Taiwan University, Taipei, Taiwan; 6 Division of Interdisciplinary Medicine and Biotechnology, Beth Israel Deaconess Medical Center/Harvard Medical School, Boston, Massachusetts, USA

## Abstract

Heart rhythm complexity analysis has been shown to have good prognostic power in patients with cardiovascular disease. The aim of this study was to analyze serial changes in heart rhythm complexity from the acute to chronic phase of acute myocardial infarction (MI). We prospectively enrolled 27 patients with anterior wall ST segment elevation myocardial infarction (STEMI) and 42 control subjects. In detrended fluctuation analysis (DFA), the patients had significantly lower DFAα2 in the acute stage (within 72 hours) and lower DFAα1 at 3 months and 12 months after MI. In multiscale entropy (MSE) analysis, the patients had a lower slope 5 in the acute stage, which then gradually increased during the follow-up period. The areas under the MSE curves for scale 1 to 5 (area 1–5) and 6 to 20 (area 6–20) were lower throughout the chronic stage. Area 6–20 had the greatest discriminatory power to differentiate the post-MI patients (at 1 year) from the controls. In both the net reclassification improvement and integrated discrimination improvement models, MSE parameters significantly improved the discriminatory power of the linear parameters to differentiate the post-MI patients from the controls. In conclusion, the patients with STEMI had serial changes in cardiac complexity.

Acute myocardial infarction (AMI) is one of the leading causes of mortality and morbidity worldwide. After the acute phase of myocardial infarction (MI), neurohormonal and mechanical stimuli lead to progressive left ventricular (LV) remodeling^1^. Thus, post-MI LV remodeling is an arrhythmic complication and it is associated with post-MI heart failure and the long-term prognosis of MI^2^,^3^.

Analysis of variations in heart rate oscillation, also known as heart rate variability (HRV), is commonly used to assess the autonomic nervous system due to its simple and noninvasive approach^4^. HRV has also been used to predict the outcomes of patients with cardiovascular disease^5^. In recent years, newer methods of heart rhythm complexity analysis have been developed based on non-linear signal modeling^6^. The principle of heart rhythm complexity analysis is based on the assumption that a healthy system exerts complex control over time to maintain operation in an ever-changing environment^7^,^8^. In a living system, many physiological signals (e.g. heart rate) exhibit irregular fluctuations which are neither random nor rigidly regular under normal conditions^9^. In contrast, these fluctuations exert a complex and dynamic pattern over multiple time scales. The complexity of physiological signal fluctuations is thought to be due to system adaptability in response to internal and external changes, and is one of the most important characteristics of a healthy living system^6^.

In heart rate dynamics, several non-linear methods have been proposed to quantify complexity. Peng and coworkers introduced detrended fluctuation analysis (DFA), which can be used to estimate whether fluctuations in small time scales are statistically equivalent to those in large time scales (called self-similarity). When heart rhythm is observed over longer periods of time, fluctuations in stride intervals exhibit long-range, fractal like correlations^10^ which may be due to the influence of multiple control systems. DFA is derived from fractal analysis and is suitable for scale-invariant signals^11^. Compared to conventional methods (e.g. spectral analysis), DFA not only allows for the detection of long-range power-law correlations embedded in a seemingly non-stationary time series, but also avoids the false detection of apparent long-range correlations that are an artifact of non-stationarity^10^. The DFA method has been successfully applied in heart rhythm research^12^,^13^. In addition, complexity in heart rate dynamics can be also estimated by entropy. Approximate entropy and sample entropy were first used to quantify changing complexity by calculating the rate of new information generation^14^,^15^. Later, taking into account the multiple time scales in physical systems, Costa *et al*. introduced the multiscale entropy (MSE) approach to represent the complexity in a system^8^. MSE quantifies whether the richness of information content (certain degree of unpredictability) can be maintained over different scales. Since its introduction, MSE has become an important tool to quantify signal complexity^16^. MSE has been widely applied in various diseases such as sepsis, heart failure, stroke, primary aldosteronism and critical illnesses which cause the “breakdown” of complexity in heart rate dynamics to capture the picture of changes in complexity^17^,^18^,^19^,^20^,^21^. In recent years, several refinements and adjustments of entropy or MSE have been introduced. For instance, fuzzy entropy uses the soft and continuous boundaries of fuzzy functions to measure physiological signals which include unpredictable and irregular properties^22^. Valencia *et al*. proposed the refined MSE that can improve the complexity evaluation^23^. The refined MSE approach uses a coarse graining method to reduce the signal of variance and apply a finite-impulse response filter to remove the fast temporal scale. Furthermore, in order to measure the complexity of data over a short period of time, short-term MSE and distribution entropy have shown advantages in small data series^24^,^25^. However, in general, DFA and MSE are the most commonly used non-linear methods to quantify complexity in heart rate dynamics in clinical research^17^,^21^,^26^.

Compared to traditional HRV parameters, heart rhythm complexity analysis has been shown to have a better prognostic power in patients with cardiovascular disease^21^,^27^. In patients with heart failure, both DFA and MSE have been shown to be useful in predicting survival^21^,^27^,^28^. In addition, DFAα1 was shown to be an important predictor of prognosis in two large clinical studies^27^,^28^. In the DIAMOND-CHF trial, after adjusting for clinical parameters, DFAα1 but not linear parameters remained an independent predictor of mortality^27^. Furthermore, in our previous study, we showed that MSE had a better prognostic power than other linear and non-linear parameters including DFAα1^21^.

In patients with MI, DFA has also been shown to provide useful information on survival and to predict ventricular tachyarrhythmia in patients with AMI, and post-MI survival in those with depressed LV function^29^,^30^,^31^. However, data on MSE in patients with AMI are lacking. In addition, LV remodeling occurs after the acute phase of MI, which may then lead to heart failure^1^. It would therefore be interesting to know when changes in heart rhythm complexity occur and the associated factors. In addition, a study investigating serial changes in heart rhythm complexity may help to elucidate the mechanism by which heart rhythm complexity occurs. Furthermore, unrecognized MI or silent MI is not uncommon and can be hard to detect^32^. If heart rhythm complexity damage persists after an MI attack, it may serve as a tool to detect silent MI. Therefore, the aims of this study were to: (1) analyze a series of heart rhythm complexity changes from the acute to chronic phase of AMI; (2) investigate the factors potentially associated with changes in heart rhythm complexity; and (3) test the value of heart rhythm complexity as a tool to detect post-MI patients.

## Results

### Patient characteristics

A total of 27 patients with ST-segment elevation myocardial infarction (STEMI) and 42 control subjects were enrolled in this study (Table 1). The STEMI patients had significantly higher levels of triglycerides and fasting glucose than the controls, however there were no significant differences in age, gender, or levels of serum creatinine, total cholesterol, low-density lipoprotein and high-density lipoprotein. During the study period, none of the STEMI patients died or required un-scheduled admissions, and none developed heart failure symptoms.

### Serial changes in echocardiographic data and plasma biomarkers in the STEMI patients

The patients had a higher LV end-systolic volume index and lower LV ejection fraction (LVEF) than the controls from day 1 to 1 year after MI, with a median LVEF of 55% at 1 year after MI (Table 2). The LV end-diastolic volume index of the patients was higher from 3 months to 1 year after MI. With regards to biomarkers, the patients had significantly higher levels of tissue inhibitor of metalloproteinase-1 (TIMP-1) and N-terminal pro-brain natriuretic peptide (NT-proBNP) on day 3 after MI than the controls. However, this difference became insignificant at 3 months, 6 months and 1 year after MI (Tables 2 and 3).

### Linear analysis of the study subjects

Seven conventional HRV metrics were evaluated. The patients had a significantly lower mean NN, standard deviation of normal to normal R peak (SDNN), and low frequency (LF) than the controls in the acute stage. However, there were no significant differences between the patients and controls at 3 months, 6 months and 1 year after MI. There were no significant differences in the other four parameters between the controls and patients at any stage (Table 4).

### Nonlinear analysis of the study subjects

With regards to the variables associated with DFA, the fractal correlation of the long-term (α2) exponent, expressed in the acute stage, was significantly lower in the patients compared to the controls, but not in the later chronic stage (3 months, 6 months and 1 year after MI). The fractal correlation of the short-term (α1) was significantly lower at 3 months and 1 year, but the short-term (α1) was not significantly different during the acute stage and 6 months after MI (TAble 4).

**Figure 1:**
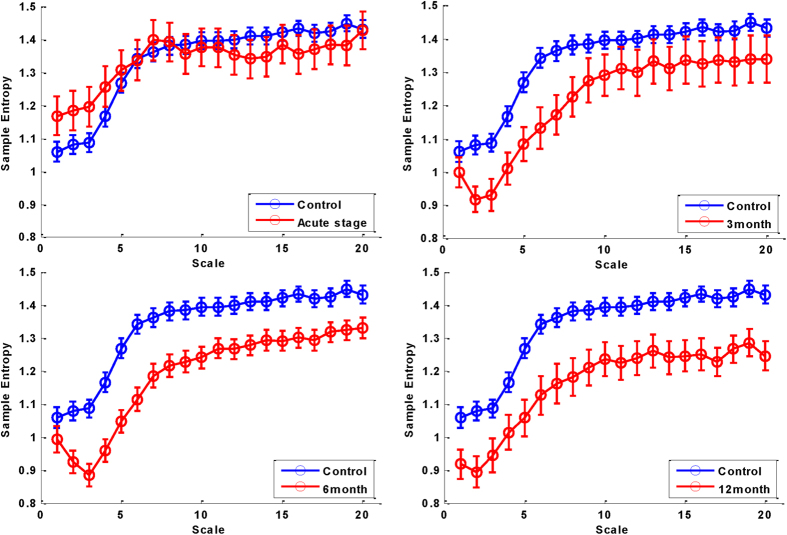
MSE in the control and STEMI patients at different time points. (**A**) Acute stage of MI, (**B**) 3 months after MI, (**C**) 6 months after MI, (**D**) 1 year after MI. Data are expressed as median (circle) and standard error of the mean (bar).

We then visually inspected all of the MSE curves, which represented the complexity in short and long time scales (Fig. 1). In the chronic stage (3 months, 6 months and 1 year after MI), all of the average MSE curves had a pattern of an initial decrease. In the acute stage, the patients had significantly lower slopes 1–5 than the controls, and this difference remained significant at 3 and 6 months after MI, becoming insignificant at 1 year after MI. The patients had significantly lower area 1–5 and area 6–20 than the controls at 3 months, 6, months and 1 year after MI.

### Comparisons of all linear and non-linear parameters to differentiate the asymptomatic post-MI patients from the controls

**Figure 2:**
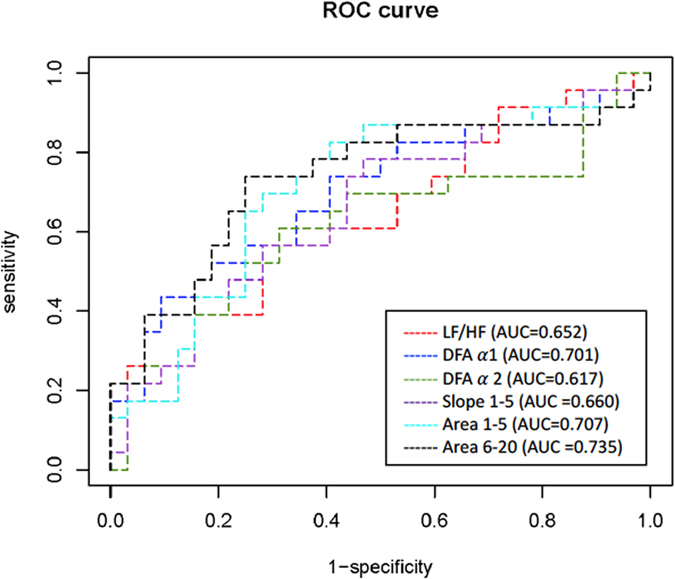
Analysis of the power of linear and non-linear parameters to discriminate STEMI patients (at 1 year) from the controls by receiver operating characteristic curve analysis. Only areas under the curve (AUC) > 0.6 were included in this figure.

To test the ability of the linear and non-linear parameters to detect the post-MI patients (1 year after MI) from the controls, we performed receiver operating characteristic (ROC) curve analysis, in which area 6–20 had the greatest discriminatory power for the two groups compared to all other linear and non-linear parameters. The areas under the curves (AUC) of mean NN, SDNN, percentage of absolute differences in normal RR intervals greater than 20 ms (pNN20), percentage of absolute differences in normal RR intervals greater than 50 ms (pNN50), LF, high frequency (HF), LF/HF, DFAα1, DFAα2, slope 1–5, area 1–5, and area 6–20, were 0.529, 0.546, 0.519, 0.586, 0.484, 0.589, 0.652, 0.701, 0.617, 0.660, 0.707, and 0.735, respectively. Figure 2 shows the ROC curves of parameters with an AUC larger than 0.6.

### The advantage of adding non-linear parameters to linear parameters to discriminate the asymptomatic post-MI patients from the controls

In both net reclassification improvement (NRI) and integrated discrimination improvement (IDI) models, DFAα1, area 1–5, and area 6–20 significantly improved the discriminatory power of SDRR, pNN20, pNN50, LF, HF, and the ratio between LF and HF components (LF/HF). Slope 1–5 significantly improved the discriminatory power of pNN20 and LF in the NRI model (Table 5).

## Discussion

There are five major findings of this study. First, in linear analysis, the STEMI patients had a significantly lower SDNN in the acute stage but not in the chronic stage. Second, in DFA, the patients had a significantly lower DFAα2 in the acute stage and lower DFAα1 at 3 months and 12 months after MI. Third, in MSE, the patients had smaller 1–5 and 6–20 areas throughout the chronic stage but not in the acute stage. In addition, the patients had a lower slope 1–5 in the acute stage which then gradually increased over the follow-up period. Fourth, area 6–20 had the greatest discriminatory power to detect the post-MI patients. Fifth, the cardiac complexity parameters improved the discriminatory power of the linear parameters to differentiate the post-MI patients from the controls.

In this study, we investigated serial changes in heart rhythm complexity in patients with MI using MSE and DFA. MSE was specifically developed to analyze heterogeneous complexity, and it has been shown to be able to extend the traditional entropy algorithm to quantify the richness of information over multiple time scales in physiological systems in recent years^33^. However, such complex structures have been shown to “break down” in patients with heart failure and other critical illnesses, and may be further affected in those with a poor prognosis^19^,^21^. In our previous studies, we found that MSE had the best prognostic power in patients with heart failure^21^ and critical illnesses requiring extracorporeal life support^19^. Furthermore, the usefulness of MSE is not limited to cardiovascular diseases, as it has also been shown to be able to predict the outcomes of patients with severe trauma requiring treatment in an intensive care unit^34^, the neurological outcomes of stroke patients^17^, and the clinical outcomes of patients with sepsis^18^. However, no previous study has reported the results of MSE analysis in patients with AMI, and to the best of our knowledge, this is the first study to use MSE to evaluate heart rhythm complexity in patients with AMI. Our results showed that the patients had significantly decreased MSE parameters including area 1–5 and area 6–20 from 3 months to 1 year after AMI. In addition, in ROC curve analysis, area 6–20 had the greatest discriminatory power to differentiate the post-MI patients from the controls. Moreover, the MSE parameters (area 1–5 and area 6–20) significantly improved the prognostic power of SDRR, pNN20, and pNN50 in both the NRI and IDI models. This demonstrates the additive effect of MSE parameters on traditional linear parameters to differentiate post-MI patients from normal subjects.

After the acute phase of MI, LV remodeling stress occurs under a series of neurohormonal and mechanical stimuli^35^, and post-MI LV stress and remodeling are the major determinants of post-MI heart failure and long-term prognosis^2^,^3^. In the present study, LV remodeling was demonstrated by increased LV chambers. In serial echocardiography, the LV end-diastolic volume index increased significantly from 3 months after MI and then throughout the follow-up period. In LV function analysis, the patients had a significantly lower LVEF than the controls from the acute to chronic stage. However, the median LVEF at 1 year post-MI was 55%, which was only mildly decreased. In addition, none of the patients developed heart failure symptoms during the follow-up period, and the NT-proBNP levels were comparable to the controls from 3 months after MI. Furthermore, the patients had comparable LF and LF/HF ratio to the controls in the chronic stage. These findings suggest that our patients recovered well after MI without serious complications in the clinical course, autonomic system modulation or LV function in the chronic stage. However, in MSE analysis, areas 1–5 and 6–20 significantly decreased from 3 months to 1 year post-MI, implying the close relationship between LV remodeling and changes in MSE parameters. This supports our hypothesis that MSE parameters may serve as a screening tool in clinical practice to detect post-MI patients without heart failure.

In the present study, we found different patterns of changes in DFA in the short- and long-term exponents. The patients had a significantly lower DFAα2 in the acute stage and lower DFAα1 at 3 months and 12 months after MI. In the patients, DFAα1 in the acute stage provided useful information on survival. In a sub-study of the DIAMOND MI trial, Huikuri reported that DFAα1 < 0.75 (recorded 5 to 10 days after MI) was the most powerful predictor of all-cause mortality after adjusting for other clinical parameters including age, LVEF, NYHA class, and medications^29^. In another study, Tapanainen *et al*. reported that DFAα1 < 0.65 (recorded during the first 7 days after AMI) was the most powerful predictor of mortality among AMI survivors^36^. In addition, Perkiomaki *et al*. reported that DFAα1 < 1.025 (recorded during the first 7 days after AMI) was a significant predictor of non-fatal acute coronary syndrome in patients with AMI^37^. Although a low DFAα1 in the acute stage of AMI is considered to be a poor prognostic factor, no study has compared DFA findings in such patients to normal subjects. Interestingly, in the present study, the patients had a significantly lower DFAα2 but comparable DFAα1 to the normal controls in the acute stage with a median DFAα1 of 0.99, which is similar to a previous study by Carvalho *et al*.^38^ and also similar to AMI patients who remained alive at the end of follow-up in the DIAMOND MI study^29^. In the present study, the median peak creatine kinase and creatine kinase-MB levels were 2452 and 208 U/L, respectively. In addition, none of the patients died or developed heart failure during 1 year of follow-up. These findings suggest that most patients had a small to medium sized MI, and that all of the patients recovered uneventfully. This may explain the relatively normal DFAα1 value during the acute stage in the STEMI patients.

The DFAα1 value decreased to 0.85 at 1 year post-MI, which is close to the value in patients with heart failure^39^. To date, only one study (OAT-EP) has investigated changes in DFA between the subacute stage (3–28 days, median 12 days) and 1 year after MI. In that study, no significant changes in DFAα1 were noted between the subacute stage and 1 year in both medication and intervention groups. In the present study, DFAα1 during the acute stage was not significantly lower than that in the controls. In contrast, DFAα1 after 1 year in the patients was significantly lower than that in the controls, and the difference in DFAα1 between the acute stage and 1 year was borderline significant (p = 0.059, data not shown). The discrepancy between the two studies may be due to differences in Holter sampling time points (i.e. when the Holter signals were recorded), sampling duration, and reperfusion time and strategy. In the OAT-EP study, DFAα1 at 1 year was very close to 1 (mean 1.02 in the medical group and 1.06 in the intervention group), which is closer to normal subjects rather than patients with heart failure^27^,^38^. Serial changes in DFAα1 and their clinical implications should be confirmed by larger prospective studies with a longer follow-up period and multiple check-ups.

There are several limitations to this study. First, this is a small pilot study with multiple Holter sampling points, and the findings should be confirmed by a larger clinical study. Second, in order to avoid the effect of Bezold-Jarisch reflexes on heart rhythm complexity analysis^40^, we only enrolled STEMI patients with anterior wall infarction in which the infarct-related artery was ultimately the left anterior descending artery. Therefore, the results cannot be applied to STEMI patients with right coronary artery or left circumflex occlusion. Third, possible confounding factors such as different levels of physical activity during the recording period^41^, or different recording periods chosen for analysis among the study subjects may also have affected the results. Of note, the STEMI patients usually had strict restrictions on physical activity (they were mostly required to remain in bed) during the acute stage, whereas there were no such restrictions in physical activity in the patients with other stages of STEMI or in the control group. This is a potential confounding factor. Fourth, because of the limited number of participants in this small study, we only used some linear and non-linear parameters. In addition, several refinements and adjustments of entropy or MSE have been introduced in recent years, such as fuzzy approximate entropy, distribution entropy, refined composite MSE, modified MSE, multi-scale symbolic entropy, and generalized MSE^16^,^19^,^22^,^23^,^25^,^42^,^43^,^44^,^45^. A larger study may be needed to investigate and compare the usefulness of other linear parameters, non-linear parameters, and new MSE refinements. Fifth, we found a V-shape in shorter MSE scales in the chronic stage in STEMI patients. The linear slope (slope 1–5) may not be the best model for this curve.

In conclusion, the patients with STEMI had different changes in cardiac complexity in the acute and chronic stages. Cardiac complexity parameters are useful to detect post-MI patients without heart failure.

## Methods

### Patients

We enrolled patients with a first STEMI who were treated within 12 hours after the onset of symptoms and who received a primary percutaneous coronary intervention. Another group of patients who were admitted for coronary angiography with no signs of significant stenosis were enrolled as the controls. Patients with a history of chronic atrial fibrillation, sick sinus syndrome, high degree conduction block, cardiovascular diseases including coronary artery disease, myocardial infarction, heart failure, cerebrovascular events, or peripheral artery disease were excluded.

In the patients, echocardiographic studies were performed at day 1 (first 24 hours after symptom onset), 3 months post-MI (90 ± 14 days after MI), 6 months post-MI (180 ± 28 days after MI), and 1 year post-MI (365 ± 28 days after MI). Blood samples were taken on day 3 (48–72 hours after symptom onset), 3 months post-MI (90 ± 14 days after MI), 6 months post-MI (180 ± 28 days after MI) and 1 year post-MI (365 ± 28 days after MI). In the control group, blood samples were taken on the morning of the coronary angiogram check-up, and echocardiographic studies were performed during the admission.

All of the patients underwent 24-h ambulatory ECG Holter recording (MyECG E3-80, Microstar Company, Taipei) during the acute stage (within 72 hours after symptom onset), 3 months post-MI (90 ± 14 days after MI), 6 months (180 ± 28 days after MI), and 1 year (365 ± 28 days after MI). The control group underwent 24-h ambulatory ECG Holter recording on the day before angiography.

This study was approved by the Institutional Review Board of National Taiwan University Hospital, and all subjects provided written informed consent including for storage of their information in the hospital database and usage for research. The methods of this study were carried out in accordance with the approved guidelines.

### Echocardiography

Standard transthoracic echocardiography (iE33 xMATRIX Echocardiography System, Philips, Amsterdam, Netherlands) was performed in each patient. The echocardiographic measurements included two-dimensional, M-mode and Doppler ultrasound recordings. LVEF measurements were based on an apical 4-chamber view measured using the area-length method or by M-mode via the parasternal long axis view (if there were no regional wall abnormalities) in accordance with the recommendations of the American Society of Echocardiography^46^.

### Data pre-processing

All of the patients underwent 24-h ECG monitoring with a sampling rate of 250 Hz, and the data were stored on a secure digital memory card for offline analysis on a computer. Each digitized ECG data point was annotated using an automatic algorithm, and then carefully inspected and corrected by technicians to obtain the RR intervals, and ectopic beats were interpolated by the adjacent RR intervals. Four hours of ECG data within the daytime were selected for analysis in order to avoid the confounding effects that may occur due to sleep or diurnal rhythm^47^. To avoid the interference of physical activity on the analysis, the 4-hour period of RR intervals we selected in this study was based on the following criteria: (1) between 9 AM–6 PM; and (2) without sudden increases in heart rate of more than 40 bpm within 1 minute. In linear analysis, the ECG data were calculated from 5-minute segments averaged over the entire 4-hour period. In addition, two nonlinear methods, MSE and DFA, were used to analyze the sequence of NN intervals over the entire 4-hour period of ECG data.

### Linear analysis

HRV was calculated from the 4-hour ECG data in the time and frequency domains according to the guidelines developed by the Task Force of the European Society of Cardiology^48^. In this study, we computed four time domain metrics: (1) the mean of normal to normal R peak, reflecting the mean value of RR interval time series; (2) SDNN, reflecting the overall magnitude of variability; (3) pNN20, the percentage of absolute differences in normal RR intervals greater than 20 ms; (4) pNN50, the percentage of absolute differences in normal RR intervals greater than 50 ms. pNN20 and pNN50 were used to evaluate the function of the autonomic system^49^. The frequency domain metrics were included in band-HF (0.15–0.4 Hz), LF (0.04–0.15 Hz), and LF/HF, which were calculated using the average power spectrum.

### Nonlinear methods

Two non-linear methods, MSE and DFA, were used to probe the fundamental characteristics of the ECG data. Both MSE and DFA can be used to quantify heartbeat complexity and variability in heartbeat time series.

### MSE

**Figure 3:**
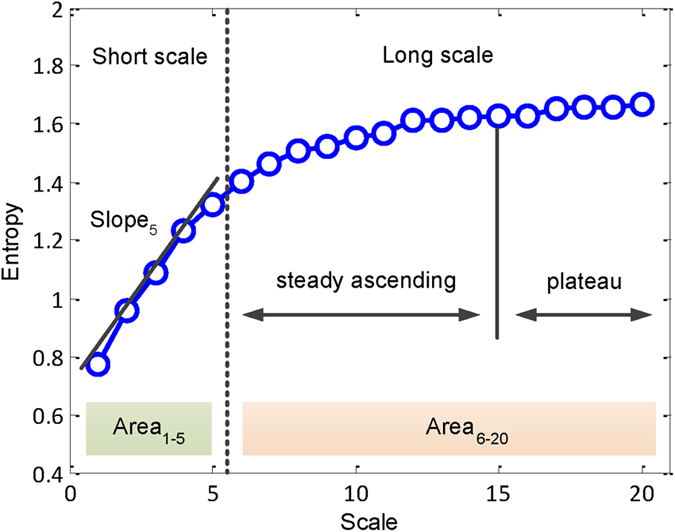
Quantification of MSE: Summation of the entropy over different scales was used to quantify the complexity over certain time scales. The common profile of entropy gradually increased as the time scale increased and reached a plateau where information richness could be accumulated rapidly if the system responded well. Three parameters of the MSE were assessed: (1) the linear-fitted slope between scales 1–5 (slope 1–5); (2) the area under the curve between scales 1–5 (area 1–5), which was used to represent complexity between short scales; (3) the area under curve between scales 6–20 (area 6–20), which was used to represent complexity between long scales.

MSE analysis can be used to estimate the physiological signals in different time scales and evaluate the predictability of a time series^33^. The MSE analysis consisted of two steps: (1) coarse-graining of the signals into different time scales; (2) quantifying the degree of irregularity in each coarse-grained time series using sample entropy^50^. In order to construct a coarse-grained series to analyze time series data, the data were first divided into *N*/*τ* non-overlapping windows of length *τ*. Finally, the data points within each window were averaged. For a given time series {1 ≤ *j* ≤ *N*}, the coarse-grained series was calculated as follows:


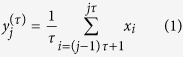


where *τ* is the scale factor and 1 ≤ *j* ≤ *N*/*τ*. In addition, the complex pattern of a time series was divided into long- and short-term curves. In this study, the different scales were useful for clinical categorization and the MSE curve up to a scale factor of 20. This scale factor was then divided into two types of parameters: (1) the first five scales were defined as the short-fitted slope (slope 1–5); and (2) the first five scales were defined as a short-fitted area (areas 1–5), and the final 15 scales were defined as the long-fitted area (area 6–20, Fig. 3). Although MSE analysis can be applied to physiological signals, the entropy values are sensitive to non-stationary artifacts, and especially trends. Hence, detrending can attenuate the spurious influence prior to MSE analysis^51^. In this study, we used empirical mode decomposition as an adaptive filter and eliminated the intrinsic mode functions slower than predefined time durations by matching their temporal frequency distribution (e.g. 2 hours^−1^) in the original R-R interval signals. The remaining intrinsic mode functions were summed up to reconstruct the signals without the trend or component slower than the predefined time duration^52^.

The underlying mechanism for short-term scales of RR intervals can be mainly attributed to respiratory sinus arrhythmia (RSA) modulated by the parasympathetic system. Therefore, slope 1–5 and area 1–5 of MSE were quantitative estimations of information richness on that particular mechanism. Area 1–5 probes the complexity structure of heart rate dynamics entrained by RSA, and a decreased area 1–5 of RR intervals has been reported in the elderly as well as in heart failure patients compared to young individuals in previous studies^21^,^33^. Slope 1–5 outlines the structure of heart rate dynamics, and a negative slope has been observed in heart failure and critically ill patients, indicating highly irregular but less information richness structure (i.e. uncorrelated fluctuations with the loss of feedback interactions)^8^,^19^,^21^. Similarly, the information richness structure of scales longer than RSA can also be quantified by the summation of specific scales. However, the underlying mechanisms accounting for the indexes of long-term scales are mostly unclear, since several physiological mechanisms exist beneath the time scales such as baroreflex and the hormonal system (e.g. aldosterone)^20^. It is still difficult to establish a standard to simply target one specific mechanism over a range of time-scales. Therefore, we used area 6–20 to estimate the overall long-term complexity.

### DFA

DFA can be used to evaluate the fractal correlation coefficient in a heart rate time series. In general, fluctuations in heart rate originate from interactions of regulatory mechanisms. To calculate the scaling exponents in DFA, a given time series *x*(*i*), 1 ≤ *i* ≤ *N*, should first be integrated as follows:


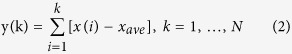


where *x*_*ave*_ is the mean of the time series *x*(*i*) and the integrated time series is divided into boxes of equal length n. Each box contains a least square line to fit the divided time series, and the time series can be detrended by removing the linear-fitted “local” trend. The root-mean-square fluctuation of the integrated time series can then be obtained as follows:


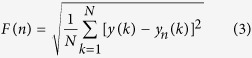


where the average fluctuations *F*(*n*) are represented as a function of the box size. The average fluctuations of the data sets are repeated in different time scales, and then a crossover phenomenon exists between the short- and long-term scales. The short-term (4–11 beats) and long-term (11–64 beats) fractal correlation exponents were calculated to obtain a clearer understanding of the fractal correlation property in a physiological system^53^. The short-term (α1) and long-term (α2) fractal correlations were calculated.

### Laboratory analysis

Serum biomarker analysis was performed in 17 STEMI patients and 37 control patients. Blood was drawn into tubes containing sodium citrate. After centrifugation, plasma was collected and stored at −80 °C until analysis. Plasma TIMP-1 was measured using an enzyme immunoassay kit (DTM100, R & D Systems, Minneapolis, USA). The intra- and inter-assay variations were <5%, and the detection limit of this method was 0.08 ng/ml. Plasma NT-proBNP was measured using an enzyme immunoassay kit (SEA485Hu, USCN Life Science, Inc., Houston, TX, USA). The intra-assay variation was <10% and inter-assay variation was <12%, and the range of detection was 39.06–2500 pg/mL.

### Statistical analysis

All of the data including the linear metrics, non-linear metrics and clinical data were expressed as median (25^th^ and 75^th^ percentiles). The ECG data and clinical characteristics of the control and STEMI groups were compared using the Mann-Whitney U test and Fisher’s exact test with relevant variables as indicated. In order to compare the ability of different Holter parameters to differentiate the STEMI patients (at 1 year after MI) from the controls, we used AUC analysis with logistic regression models. We used C-statistics to describe the discrimination of the models before and after adding non-linear parameters^54^,^55^,^56^.

NRI and IDI models were used to assess improvements in prediction using two different logistic regression models^55^. For each individual, we computed the case (STEMI patients at 1 year after MI) probability using the original model, *Po*, and the case probability using the updated model, *Pu*. The cases and controls were classified according to their *Po* and *Pu* values (Table 6).

Definitions:

 p1 = *Nss*/*Ns* (increasing rate of successfully predicting cases)

 p2 = *Nnn*/(*N* − *Ns*) (increasing rate of successfully predicting controls)

 p3 = *Nns*/*Ns* (decreasing rate of successfully predicting cases) = 1 − p1

 p4 = *Nsn*/(*N* − *Ns*) (decreasing rate of successfully predicting controls) = 1 − p2

Then NRI = p1 + p2 − p3 − p4 = 2 (p1 + p2 − 1). IDI was defined as the average improvement in case detection probability for all patients after adopting the updated model. That is,


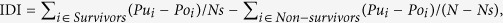


where *Po*_*i*_ and *Pu*_*i*_ were the case detecting probabilities using the original model and the updated model for the *i*th individual; *i* = 1, …, *N*.

All statistical analyses were performed using R 3.3.2 software (R Foundation for Statistical Computing, Vienna, Austria). A two-sided *p* value of less than 0.05 was considered to be statistically significant.

## Additional Information

**How to cite this article:** Chiu, H.-C. *et al*. Serial heart rhythm complexity changes in patients with anterior wall ST segment elevation myocardial infarction. *Sci. Rep.* **7**, 43507; doi: 10.1038/srep43507 (2017).

**Publisher's note:** Springer Nature remains neutral with regard to jurisdictional claims in published maps and institutional affiliations.

## Figures and Tables

**Table 1:** Demographic data of the patients and controls.

	Controls (n = 42)	STEMI patients (n = 27)	p value
Age, years	58 (53; 63)	63 (55; 68)	0.091
Male, n (%)	36 (85)	25 (92)	0.468
Hypertension, n (%)	27 (64)	16 (59)	0.799
Diabetes, n (%)	7 (17)	4 (15)	1.000
Total cholesterol, mg/dl	178 (161; 202)	166 (147; 187)	0.184
Triglycerides, mg/dl	122 (84.5; 153.5)	76 (43; 118)	0.005
LDL, mg/dl	108 (75; 136)	110 (92.5; 125)	0.629
HDL, mg/dl	45 (40; 51)	36 (34; 47)	0.148
Creatinine, mg/dL	1.0 (0.9; 1.1)	0.9 (0.8; 1.1)	0.156
Fasting glucose, mg/dl	97 (91; 107.5)	120 (75; 170)	0.002
Peak CK, U/L	—	2452 (1323; 4617)	
Peak CK-MB, U/L	—	208 (137 341)	

Values are median (25^th^; 75^th^ percentile). Abbreviations: STEMI = ST segment elevation myocardial infarction; LDL = Low-density lipoprotein; HDL = High-density lipoprotein.

**Table 2:** Echocardiographic data of the patients and controls.

	Controls	STEMI patients			
		Day 1	3^rd^ month	6^th^ month	12^th^ month
IVST, mm	11 (9.7; 13)	11 (8.6; 12.5)	11 (9.5; 13)	11 (9.3; 13.5)	10 (9.1; 12)
		P = 0.644	P = 0.949	P = 0.541	P = 0.059
LVPWT, mm	11 (9.5; 12)	11 (7.9; 12)	11 (9.3; 12)	10 (9.1; 11)	10 (9.1; 11)
		P = 0.858	P = 1.000	P = 0.127	P = 0.182
LVEDD, mm	47 (45; 50)	42 (39; 48)	50 (45.5; 54)	50 (45.5; 553)	50 (45.5; 52.5)
		P = 0.034	P = 0.100	P = 0.079	P = 0.022
LVESD, mm	28 (25; 31)	29 (25.5; 34)	33 (29; 37)	34 (28.5; 38)	34 (29; 38)
		P = 0.122	P < 0.001	P < 0.001	P < 0.001
LVEDVI, mm^3^/m^2^	55 (47; 59.)	48 (39; 62)	68 (51; 77)	68 (56; 73)	68 (58; 74)
		P = 0.283	P = 0.009	P = 0.002	P < 0.001
LVESVI, mm^3^/m^2^	16 (13; 20)	21 (16.; 27)	26 (19; 35)	27. (20; 33)	30 (19; 35)
		P < 0.001	P < 0.001	P < 0.001	P < 0.001
LVEF, %	70 (66; 77)	53 (44; 61)	55 (52; 58)	54 (51; 59)	55 (50; 61)
		P < 0.001	P < 0.001	P < 0.001	P < 0.001
E wave, cm/sec	75 (59; 84)	66 (54; 86)	76 (63; 84)	71 (60; 78)	68 (55; 75)
		P = 0.159	P = 0.913	P = 0.326	P = 0.044
A wave, cm/sec	86 (73; 94)	70 (59; 85)	83 (65; 99)	85.9 (61.6; 96.5)	88 (70; 100)
		P = 0.015	P = 0.689	P = 0.757	P = 0.772
DT, ms	195 (160; 250)	210 (150; 235)	210 (165; 240)	210 (170; 225)	210 (170; 260)
		P = 0.962	P = 0.507	P = 0.729	P = 0.402

Values are median (25^th^; 75^th^ percentile). *p* value: the data in each stage of the STEMI group compared to the control group individually. Abbreviations: STEMI = ST segment elevation myocardial infarction; IVS = interventricular septal thickness; LVPWT = left ventricular posterior wall thickness; LVEDD = left ventricular end-diastolic diameter; LVESD = left ventricular end-systolic diameter; LVEDVI = left ventricular end-diastolic volume index; LVESVI = left ventricular end-systolic volume index; LVEF = left ventricular ejection fraction; DT = deceleration time.

**Table 3:** Biomarker data.

	Controls	STEMI patients			
		D3	3^rd^ month	6^th^ month	12^th^ month
TIMP-1, ng/mL	77 (62; 87)	101 (71; 138)	95 (68; 111)	96 (60; 113)	91 (31; 151)
		P = 0.013	P = 0.097	P = 0.109	P = 0.918
NT-proBNP, pg/ml	251 (88; 389)	423 (191; 810)	301 (101; 490)	310 (135; 402)	321 (112; 392)
		P = 0.032	P = 0.416	P = 0.223	P = 0.479

Values are median (25^th^; 75^th^ percentile). *p* value: the data in each stage of the STEMI group compared to the control group individually. Abbreviations: STEMI = ST segment elevation myocardial infarction; NT-proBNP = N-terminal of pro-brain natriuretic peptide; TIMP = tissue inhibitor of metalloproteinase.

**Table 4:** Holter data of the patients and controls.

	Controls	STEMI patients			
		acute stage	3^rd^ month	6^th^ month	12^th^ month
**Time domain analysis**					
Mean NN, ms	823 (746; 903)	725 (684; 838)	763 (691, 837)	789 (710; 879)	789 (697; 902)
		0.012	0.156	0.602	0.562
SDNN, ms	85 (73; 107)	52 (42; 73)	84 (62; 99)	81 (66; 101)	91 (63; 125)
		<0.001	0.282	0.210	0.994
pNN20, %	30.1 (18.9; 40.7)	22.3 (15.4; 38.3)	23.6 (14.8; 40.4)	21.1 (16.3; 37.9)	31.1 (12.1; 40.2)
		0.268	0.277	0.266	0.718
pNN50, %	3.1 (1.6; 7.7)	2.0 (1.4; 6.8)	3.7 (1.2; 6.6)	2.9 (1.1; 6.6)	4.6 (1.8; 9.6)
		0.402	0.806	0.518	0.465
**Frequency domain analysis**					
LF	199 (123; 288)	108 (71; 260)	201 (68; 265)	147 (54; 299)	168 (90; 369)
		0.020	0.343	0.128	0.688
HF	98 (51; 209)	74 (45; 148)	146 (40; 202)	96 (38; 190)	137 (74; 248)
		0.349	0.692	0.440	0.638
LF/HF	1.52 (1.10; 3.36)	1.55 (1.04; 2.78)	1.42 (0.98; 2.01)	1.68 (1.24; 2.17)	1.30 (0.95; 2.14)
		0.409	0.180	0.665	0.068
**Detrended fractal analysis**					
Alpha-1	0.99 (0.85; 1.20)	0.99 (0.81; 1.10)	0.90 (0.81; 0.98)	0.98 (0.89; 1.10)	0.85 (0.780; 1.01)
		0.343	0.041	0.702	0.032
Alpha-2	1.08 (1.04; 1.11)	0.98 (0.89; 1.04)	1.07 (1.01; 1.11)	1.09 (1.03; 1.13)	1.11 (1.01; 1.16)
		<0.001	0.583	0.347	0.116
**Multiscale entropy**					
Slope 1–5	0.061 (0.030; 0.084)	0.019 (−0.021; 0.049)	0.032 (−0.005; 0.060)	0.034 (−0.015; 0.054)	0.031 (−0.017; 0.057)
		0.002	0.011	0.018	0.002
Area 1–5	4.54 (3.90; 4.93)	4.93 (3.54; 5.68)	3.97 (3.09; 4.36)	3.82 (3.38; 4.52)	3.98 (3.59; 4.23)
		0.375	0.002	0.003	0.008
Area 6–20	19.6 (18.3; 21.3)	18.9 (17.1; 23.3)	18.3 (15.1; 19.7)	17.6 (16.1; 19.7)	17.3 (15.6; 19.0)
		0.936	0.011	0.003	0.001

Values are median (25^th^; 75^th^ percentile). *p* value: the data in each stage of the STEMI group compared to the control group individually. Abbreviations: STEMI = ST segment elevation myocardial infarction; STEMI = ST segment elevation myocardial infarction; SDNN = standard deviation of normal RR intervals; NN20 = percentage of the absolute change in consecutive normal RR interval exceeds 20 ms; pNN50 = percentage of the absolute change in consecutive normal RR interval exceeds 50 ms; AUC = area under the curve; NRI = net reclassification improvement; IDI = integrated discrimination improvement; MSE = multiscale entropy; LF = low frequency; HF = high frequency.

**Table 5:** AUC, NRI, and IDI models of linear parameters before and after adding non-linear parameters to differentiate post-STEMI patients (1 year) from controls.

	AUC	P value	NRI	P value	IDI	P value
SDNN	0.546					
+slope 1–5	0.658	0.239	0.38	0.156	0.058	0.066
+area 1–5	0.713	0.087	0.815	<0.001	0.090	0.017
+area 6–20	0.734	0.045	0.679	0.008	0.136	0.004
+DFA α1	0.713	0.084	0.579	0.025	0.112	0.009
pNN20	0.519					
+slope 1–5	0.642	0.279	0.592	0.023	0.059	0.071
+area 1–5	0.735	0.047	0.878	<0.001	0.102	0.011
+area 6–20	0.732	0.047	0.853	<0.001	0.143	0.003
+DFA α1	0.701	0.111	0.516	0.040	0.112	0.009
pNN50	0.586					
+slope 1–5	0.657	0.468	0.380	0.156	0.058	0.069
+area 1–5	0.717	0.167	0.666	0.008	0.097	0.012
+area 6–20	0.735	0.113	0.853	<0.001	0.141	0.002
+DFA α1	0.701	0.231	0.516	0.047	0.108	0.011
LF	0.483					
+slope 1–5	0.654	0.553	0.554	0.030	0.059	0.067
+area 1–5	0.705	0.229	0.666	0.008	0.093	0.015
+area 6–20	0.733	0.125	0.853	<0.001	0.141	0.002
+Alpha-1	0.701	0.212	0.516	0.047	0.111	0.009
HF	0.588					
+slope 1–5	0.657	0.522	0.443	0.096	0.059	0.067
+area 1–5	0.697	0.290	0.815	<0.001	0.093	0.016
+area 6–20	0.732	0.146	0.729	0.003	0.140	0.003
+Alpha-1	0.706	0.227	0.617	0.017	0.114	0.009
LF/HF	0.652					
+slope 1–5	0.669	0.799	0.318	0.239	0.034	0.152
+area 1–5	0.706	0.410	0.554	0.034	0.064	0.029
+area 6–20	0.728	0.254	0.492	0.062	0.109	0.005
+DFA α1	0.732	0.264	0.766	0.002	0.122	0.006

Abbreviations: STEMI = ST segment elevation myocardial infarction; SDNN = standard deviation of normal RR intervals; pNN20 = percentage of the absolute change in consecutive normal RR interval exceeds 20 ms; pNN50 = percentage of the absolute change in consecutive normal RR interval exceeds 50 ms; AUC = area under the curve; NRI = net reclassification improvement; IDI = integrated discrimination improvement; MSE = multiscale entropy; LF = low frequency; HF = high frequency.

**Table 6:** Illustration of NRI and IDI computations.

	Truth	
Occasions	case	control
*Po* < *Pu*	*Nss*	*Nsn*
*Po* >= *Pu*	*Nns*	*Nnn*
Total	*Ns*	*N* − *Ns*

The survival probabilities using the original model and the updated model are denoted as *Po* and *Pu*, respectively. The numbers of survivors and non-survivors were shown in the table according to their *Po* and *Pu* values. Defining p1 = *Nss*/*Ns* (increasing rate of successfully predicting survivors), p2 = *Nnn*/(*N* − *Ns*) (increasing rate of successfully predicting non-survivors), p3 = 1 − p1, and p4 = 1 − p2. Then NRI = p1 + p2 − p3 − p4 = 2(p1 + p2 − 1). The IDI was defined as the average improvement in survival probability for all patients after adopting the updated model: 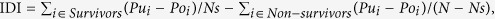 where *Poi* and *Pui* were the survival probabilities using the original model and the updated model for the *i*th individual; *i* = 1, …, *N*.
